# Development of Eczema Vaccinatum in Atopic Mouse Models and Efficacy of MVA Vaccination against Lethal Poxviral Infection

**DOI:** 10.1371/journal.pone.0114374

**Published:** 2014-12-08

**Authors:** Jarmila Knitlova, Vera Hajkova, Ludek Voska, Jana Elsterova, Barbora Obrova, Zora Melkova

**Affiliations:** 1 Department of Immunology and Microbiology, 1st Medical Faculty, Charles University, Studnickova 7, 128 00, Prague 2, Czech Republic; 2 Department of Clinical and Transplant Pathology, Institute for Clinical and Experimental Medicine, Videnska 9, 140 21, Prague 4, Czech Republic; University of Georgia, United States of America

## Abstract

Smallpox vaccine based on live, replicating vaccinia virus (VACV) is associated with several potentially serious and deadly complications. Consequently, a new generation of vaccine based on non-replicating Modified vaccinia virus Ankara (MVA) has been under clinical development. MVA seems to induce good immune responses in blood tests, but it is impossible to test its efficacy in vivo in human. One of the serious complications of the replicating vaccine is eczema vaccinatum (EV) occurring in individuals with atopic dermatitis (AD), thus excluding them from all preventive vaccination schemes. In this study, we first characterized and compared development of eczema vaccinatum in different mouse strains. Nc/Nga, Balb/c and C57Bl/6J mice were epicutaneously sensitized with ovalbumin (OVA) or saline control to induce signs of atopic dermatitis and subsequently trans-dermally (t.d.) immunized with VACV strain Western Reserve (WR). Large primary lesions occurred in both mock- and OVA-sensitized Nc/Nga mice, while they remained small in Balb/c and C57Bl/6J mice. Satellite lesions developed in both mock- and OVA-sensitized Nc/Nga and in OVA-sensitized Balb/c mice with the rate 40–50%. Presence of mastocytes and eosinophils was the highest in Nc/Nga mice. Consequently, we have chosen Nc/Nga mice as a model of AD/EV and tested efficacy of MVA and Dryvax vaccinations against a lethal intra-nasal (i.n.) challenge with WR, the surrogate of smallpox. Inoculation of MVA intra-muscularly (i.m.) or t.d. resulted in no lesions, while inoculation of Dryvax t.d. yielded large primary and many satellite lesions similar to WR. Eighty three and 92% of mice vaccinated with a single dose of MVA i.m. or t.d., respectively, survived a lethal i.n. challenge with WR without any serious illness, while all Dryvax-vaccinated animals survived. This is the first formal prove of protective immunity against a lethal poxvirus challenge induced by vaccination with MVA in an atopic organism.

## Introduction

Vaccinia virus (VACV) from the Poxvirus family has been used as a live vaccine against smallpox. A worldwide vaccination campaign using various strains of VACV led to the eradication of this life-threatening disease. Consequently, vaccination of the general population has been stopped. At the beginning of this century due to concerns about the smallpox misuse in a bioterrorist attack, vaccination against smallpox was re-introduced [1]. Original stocks of VACV strain Dryvax as well as second generation tissue culture-grown stocks (ACAM2000) have been used [2]. New, highly attenuated vaccine based on non-replicating Modified vaccinia virus Ankara (MVA; IMVAMUNE) is under clinical development and seems to induce good immune responses in blood tests and in animal models [3], [4], [5], [6], [7]. It is impossible, though, to test its efficacy in vivo in human.

Vaccination against smallpox induces good response of both cellular and humoral immunity, but it may be associated with several post-vaccination complications together with a substantial risk of spread of VACV among the contacts of vaccinees [8], [9], [10]. The most severe complications include progressive vaccinia, post-vaccination encephalitis, eczema vaccinatum and a recently described myopericarditis [8]. In this article we focused on eczema vaccinatum (EV). It occurs namely in individuals with a history of atopic dermatitis or eczema (AD), and it is caused by a dissemination of VACV in the skin apart from the vaccination site, after a self-inoculation or through a contact with a vaccinee. During the worldwide vaccination campaign, EV incidence among primary vaccinees was approximately 10–40 per million [11], while the lethality was around 1–6% [12].

Atopic dermatitis is an increasingly common inflammatory skin disease that is genetically determined but the environmental and neuropsychological factors contribute to the development of the disease also [13]. Individuals with AD develop itchy skin lesions on distinct parts of the body and roughly 70–80% of them have elevated serum IgE levels [13]. According to one hypothesis, AD is primarily caused by abnormalities of skin innate immunity, resulting in the infiltration with skin-homing Th_2_ cells during the acute phase and in complex inflammatory responses. Second hypothesis considers as a primary defect the immune dysregulation towards Th_2_ immune responses together with IgE-mediated sensitization [12], [14], [15].

Currently, there is a variety of mouse models of AD. They include models with spontaneous manifestation of AD, genetically engineered mice, models with skin lesions induced by sensitization with an antigen, and models with severe combined immunodeficiencies [16].

In our studies, we compared the effects of wild-type VACV strain Western Reserve (WR) inoculation in the skin of three different mouse strains. Namely, we used Japanese Nc/Nga mice, a spontaneously mutated inbred strain that reveals many characteristics similar to human AD. About 50% of mice develop spontaneous skin lesions in non-SPF conditions, suggesting that the epigenetic or environmental factors play a role also [17]. The mice were shown to have elevated serum levels of IgE, histopathological changes of the skin (thickening of dermis and epidermis, infiltration with eosinophils and mononuclears) and Th_2_-dominant immune responses [18]. Further, we used Balb/c and C57Bl/6 mice. Both strains were described to develop AD when sensitized epicutaneously with ovalbumin using tape-stripping [19], [20]. In general, Balb/c mice reveal Th_2_-shifted immune responses, while C57Bl/6 mice reveal Th_1_-dominant immune responses; accordingly, the individual mouse strains are more sensitive or resistant to intracellular pathogens, respectively [21].

For studies of eczema vaccinatum, both Nc/Nga and Balb/c mice sensitized with various allergens have been used [22], [23], [24], but genetically modified mice derived from C57Bl/6 strain are also commonly used to study responses to VACV. Nevertheless, different sensitization and inoculation protocols used by individual research groups make the results difficult to compare. In this paper, we show for the first time a direct comparison of all three mouse strains as an AD/EV model. Our comparison showed that Nc/Nga mice developed the condition most similar to human EV, namely forming satellite pox lesions distant from the site of WR inoculation and highest WR titer in the inoculation site. Therefore we used Nc/Nga mice as a model atopic organism to test the ability of attenuated non-replicating MVA in comparison with the replicating Dryvax to mount a protective response against the intra-nasal infection with a lethal dose of wild-type vaccinia virus strain WR, the surrogate of smallpox [25]. In this work, we clearly show that despite of the defects in skin immunity, atopic Nc/Nga mice are able to mount an effective protective immunity against the lethal i.n. challenge with VACV strain WR. To our knowledge, this is the first formal report proving efficiency of MVA immunization against a lethal poxvirus challenge in an atopic organism.

## Materials and Methods

### 1. Ethics statement

This work was carried out in accordance with European regulations for transport, housing and care of laboratory animals (Directive 2010/63/EU on the protection of animals used for scientific purposes). The animals were housed in housing facilities accredited by the Ministry of Agriculture of the Czech Republic and monitored daily. All the infectious experiments were performed in the Biosafety Level 2 laboratory with negative pressure and using HEPA-filtered animal boxes. Full details of the animal experiments, including the mortality aspects, were approved by the Experimental Animal Use Committee of the 1st Medical Faculty of Charles University and the Ministry of Education of the Czech Republic (experimental protocols No. GA UK 100307 – 208/06, 0021620806 MSM, P302/10/0083 – 298/09). These protocols also addressed the cases of unexpected mortality. All individuals working with infected animals were vaccinated with the standard smallpox vaccine in the past.

### 2. Animals and Viruses

We used male and female mice of three strains: Nc/Nga (kind gift of Riken BioResource center, Japan) and C57Bl/6J bred and reared in specific pathogen-free (SPF) conditions in The Center for Experimental Biomodels at the 1^st^ Medical Faculty, Charles University, and Balb/c purchased from Harlan. Faculty facility ensures standard SPF conditions (HEPA-filtered air, sterilized beddings and food with all manipulations in laminar flow hoods). The animals bred in the faculty facility were routinely checked by a veterinary service, while the animals purchased from Harlan were delivered with a Health Monitoring Report. They were acclimatized in conventional housing for 1 week before the start of sensitization at the age of 6–8 weeks. The animals were assigned to cages and or/experimental groups based on their sex and litter (individual littermates were split into different experimental groups) with maximum of 5 mice/cage. The weight of Nc/Nga mice was 18–20 g at the start of sensitization, and 24–30 and 26–32 g, females and males respectively, at the start of immunization and prior to the lethal challenge. Balb/c and C57Bl/6J mice weighed generally 2 g less.

VACV strain Western Reserve (WR), Dryvax and Modified vaccinia virus Ankara (MVA) were used. MVA [26] and a plaque isolate of Dryvax (D50; [27], [28], [29]) were kindly provided by Dr. Nemeckova. VACV strains WR and Dryvax were propagated in BSC-40 cells as described previously [29], [30], while MVA was propagated in primary chicken embryonal fibroblasts (CEFs; kindly provided by Dr. Geryk from the Czech Academy of Sciences). Dryvax and WR were purified by a sucrose gradient sedimentation [31], MVA by a centrifugation through a cushion of 45% sucrose. Virus titers were determined by serial dilutions and plaque assays in BSC-40 cells (Dryvax, WR) [29], [30] or in CEFs using immunodetection of plaques with a rabbit polyclonal antiserum against vaccinia virus (MVA); [32].

### 3. Epicutaneous (EC) sensitization and VACV infection

All the experimental manipulations with mice were performed in anesthesia with avertin (2,2,2,tribromoethanol in tertial amylalcohol, 1 mg/ml) administered intra-peritoneally (i.p.; 12–16 µl/g of weight). Mice in experiments were monitored daily and at the specified time points, they were sacrificed humanely under terminal anesthesia with diethyl ether and exsanguination from the jugular vein. In protective experiments, the basic aim was to assess the effect of the prior immunization on the survival of atopic animals; therefore, the lethality was expected in control non-immune mice, while the survival was anticipated in immune ones. The criteria for humane endpoint defined in the study protocol P302/10/0083 – 298/09 were weight loss under 25% and decreased body temperature. All efforts were made to minimize the suffering of the mice during manipulations with them.

**Figure 1:**
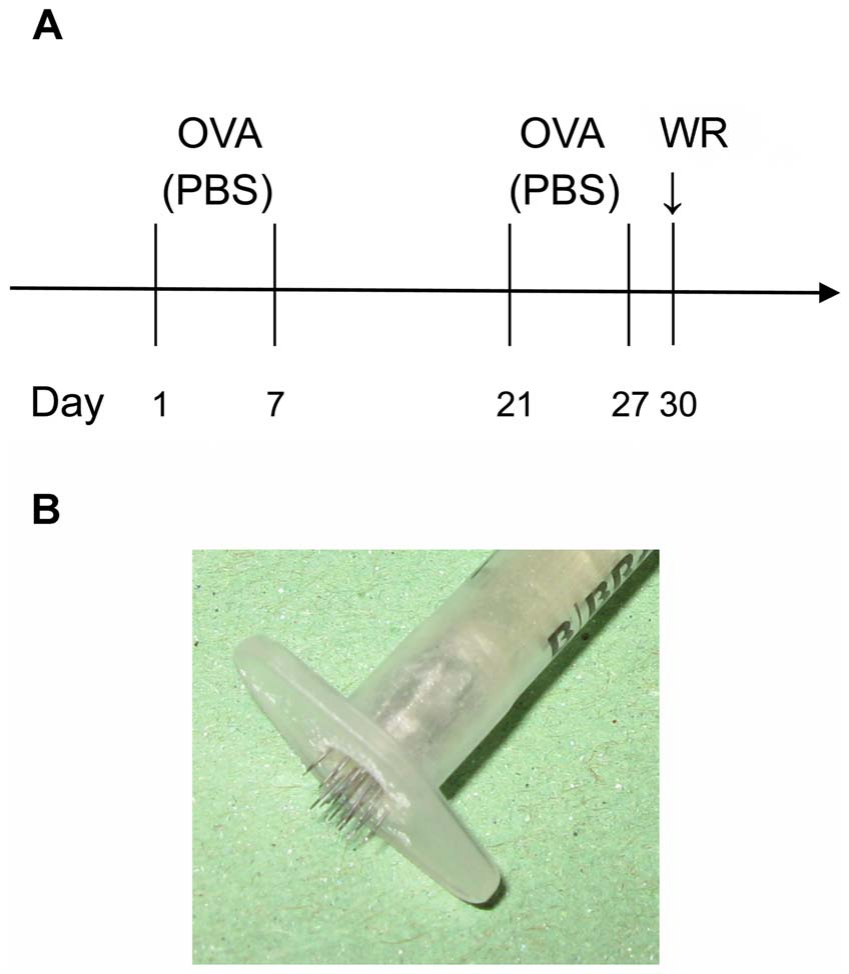
Epicutaneous sensitization and WR inoculation protocol. (A) Two cycles of sensitization with OVA or control PBS by tape-stripping and inoculation of WR using a vaccination stamp. (B) Vaccination stamp.

For experiments studying development of AD and EV, mice of all strains were anesthetized with avertin, and sensitized epicutaneously with ovalbumin (OVA; 100 µl of 1 mg/ml in PBS) or PBS only on the right side of the back using tape-stripping (TS) according to scheme in Fig. 1A [19], [20]. Three days after the end of second sensitization, the mice were anesthetized with avertin, mock-inoculated at the site of sensitization with OVA in PBS (final concentration 0.5 mg/ml) or inoculated with 1×10^7^ PFU of VACV strain WR with OVA in PBS using a vaccination stamp (trans-dermal (t.d.) inoculation). The inocula were then covered for 7 days with an impermeable bandage and the mice were humanely sacrificed 7 or 14 days after virus inoculation. For experiments studying protective effect of MVA and Dryvax immunizations, the mice were sensitized with PBS and immunized with 10^8^ PFU of MVA t.d. or intra-muscularly (i.m.) or with 10^7^ PFU of Dryvax t.d. (Fig. 1A) under anesthesia with avertin. Three weeks after the immunization, the anesthetized mice were intra-nasally (i.n.) challenged with a lethal dose of VACV strain WR, 5×10^6^ PFU in 10 µl of PBS. The infected animals were monitored and clinically scored daily as described above. All control non-immune and three immunized mice died spontaneously before meeting the humane endpoint criteria, while the surviving animals were sacrificed humanely 2 weeks after the challenge. The mice were post-mortem dissected and the weights of spleen, lungs, liver and kidneys were determined. The exact cause of death was not further explored. No additional adjustments to the approved protocol were made during the original experiments presented in this study.

The vaccination stamp (Fig. 1B) was prepared using 20 acupuncture needles (thickness 0.16 mm; HuangQiu Acupuncture Medical Appliance Co. Ltd, Suzhou, China) inserted in the outer part of a syringe, forming a circle with a diameter of 5 mm and protruding over the rim 1.5 mm. The depth of the transdermal puncture was typically 0.5–1 mm.

### 4. Titer of WR in the skin lesions

The skin lesions of infected animals were frozen, and then homogenized in ice-cold DMEM (in a volume corresponding to 4x weight of the skin lesion). After 2 cycles of freezing and thawing, the samples were sonicated and centrifuged and the supernatant was used for determination of virus titer using serial dilutions and plaque assays in BSC-40 cells [29], [30]. Similarly, WR titers were determined in lungs, liver, kidney and spleen of Nc/Nga mice that succumbed to lethal i.n. infection with WR.

### 5. Histology and histochemistry

Skin biopsies were taken 7 days after virus inoculation, fixed with 4% paraformaldehyde in PBS and embedded in paraffin. Deparaffinized sections were stained with hematoxylin and eosin and analyzed by light microscopy using camera Olympus C-5050 mounted on Olympus microscope BX 51 (Olympus Optical, Hamburg, Germany) and software Olympus DP-SOFT (Software Imaging Systems, Münster, Germany); original magnification 100x, 200x and 400x.

Mast cells were determined in multiple 4-µm skin sections using staining with 0.05% Toluidine blue in distilled water and quantified in 5–10 high power fields (original magnification 200x) for each mouse, the mean of which was used for further calculations and statistical evaluations.

### 6. Immunohistochemistry

WR proteins were detected using a primary rabbit anti-VACV antibody (ViroStat, Inc., Portland, Maine), dilution 1∶200, and secondary peroxidase-labelled polymer conjugated to goat anti-rabbit immunoglobulins, dilution 1∶200 (EnVision + System; Dako), DAB was used as a chromogenic substrate. The sections were then counterstained with hematoxylin.

### 7. Determination of serum IgG_1_ and IgG_2a_ antibodies specific for VACV and of total serum IgE antibodies

VACV-specific antibodies IgG_2a_ and IgG_1_ were determined using sandwich Elisa performed in 96 well plates (Nunc, MaxiSorp) coated with 1×10^7^ PFU/well of purified, UV-inactivated VACV strain WR (Advanced Biotechnologies and our own preparation [33]). Sera collected 14 days after VACV inoculation were diluted 1∶1,000 and 1∶5,000 for IgG_1_ and 10-times more for IgG_2a_ determinations. In protective experiments, sera were collected 3 weeks after immunization and used in dilutions 1∶50–1∶25,000 for IgG_2a_ and 1∶50–1∶2,000 for IgG_1_, and 14 days after a lethal challenge with VACV strain WR in surviving Nc/Nga mice in dilutions 1∶100,000–∶200,000 for IgG_2a_ and 1∶10,000–1∶20,000 for IgG_1_. Consequently, the plates were incubated overnight in 4°C. Bound IgG_2a_ and IgG_1_ were detected using biotinylated rat anti-mouse antibodies (0.5 and 1 µg/ml for IgG_2a_ and IgG_1_, respectively; BD Pharmingen), streptavidin coupled to HRP (0.5 µg/ml; Immunotech) and TMB (Amresco). The reaction was stopped with 1 M H_2_SO_4_ and optical density was measured at 450 nm using a plate reader Victor 1420-012 (PerkinElmer). Levels of IgG_1_ and IgG_2a_ are expressed as relative values calculated from a calibration curve of a standard. This standard was prepared from infected Nc/Nga mouse serum and was added to each Elisa test in several dilutions defined for each isotype. As the amount of individual IgG_1_ and IgG_2a_ in the standard is unknown, relative levels of IgG_1_ cannot be compared to the levels of IgG_2a_ in the tested samples. Total serum IgE antibodies were determined similarly as specific IgG's, using rat anti-mouse IgE antibody (2 µg/ml in PBS) for coating and biotinylated rat anti-mouse antibody (2 µg/ml; BD Pharmingen) for detection. Purified mouse IgE standard (BD Pharmingen) was used for the calibration curve.

### 8. Characterization of the neutralization capacity of mice sera

The total neutralizing capacities of mice sera were determined by a plaque reduction neutralization test. Sera of infected and uninfected mice (9 µl) were incubated with 1 µl of DMEM containing 8.75×10^3^ PFU of VACV strain WR (total volume 10 µl; final virus concentration 8.75×10^5^ PFU/ml) for 12 h in 37°C. The resulting virus titers were then determined using serial dilutions and plaque assays as described in section *4*. The original amount of input WR in the dilution used for determination of the number of plaques was 723 PFU/well. Efficiency of neutralization capacity was expressed as percentage of resulting plaque numbers in each group compared to the numbers in mock-inoculated Nc/Nga mice.

### 9. Statistical analysis

Data were analyzed by one-way ANOVA global test followed by LSD post-hoc comparison tests using Statistica software.

## Results

### 1. Induction of atopic dermatitis and eczema vaccinatum

At the onset of experiments, we determined the conditions for development of atopic dermatitis in three different mouse strains: in spontaneously atopic Nc/Nga mice, in Th_2_-skewed Balb/c and in Th_1_-skewed C57Bl/6 mice. In SPF environment, Nc/Nga mice did not spontaneously develop any macroscopic signs of AD and even in conventional housing, the incidence of macroscopic dermatitis-like lesions was very low. Therefore, we started to epicutaneously sensitize the mice with OVA and tape-stripping. The skin lesions in Nc/Nga mice developed already after the first sensitization, being most severe after the second one. Inclusion of a third cycle of sensitization led to a slight decline in the severity of lesions; therefore two cycles of sensitization were used in the following experiments. Interestingly, dry, scaly lesions and itchy behavior were evident predominantly in male Nc/Nga mice. In contrast, Balb/c or C57Bl/6 mice did not develop any macroscopic lesions during the sensitization with OVA. We also tested dust mites and household fungi in Nc/Nga mice, getting similar results as with OVA application (data not shown). For VACV inoculation, we used a specifically developed vaccination stamp (Fig. 1B). This stamp provides a uniform size and depth of VACV inoculation, the resulting lesions can be better quantified, and satellite lesions can be clearly distinguished.

#### 1.1. Histological analysis of skin samples

**Figure 2:**
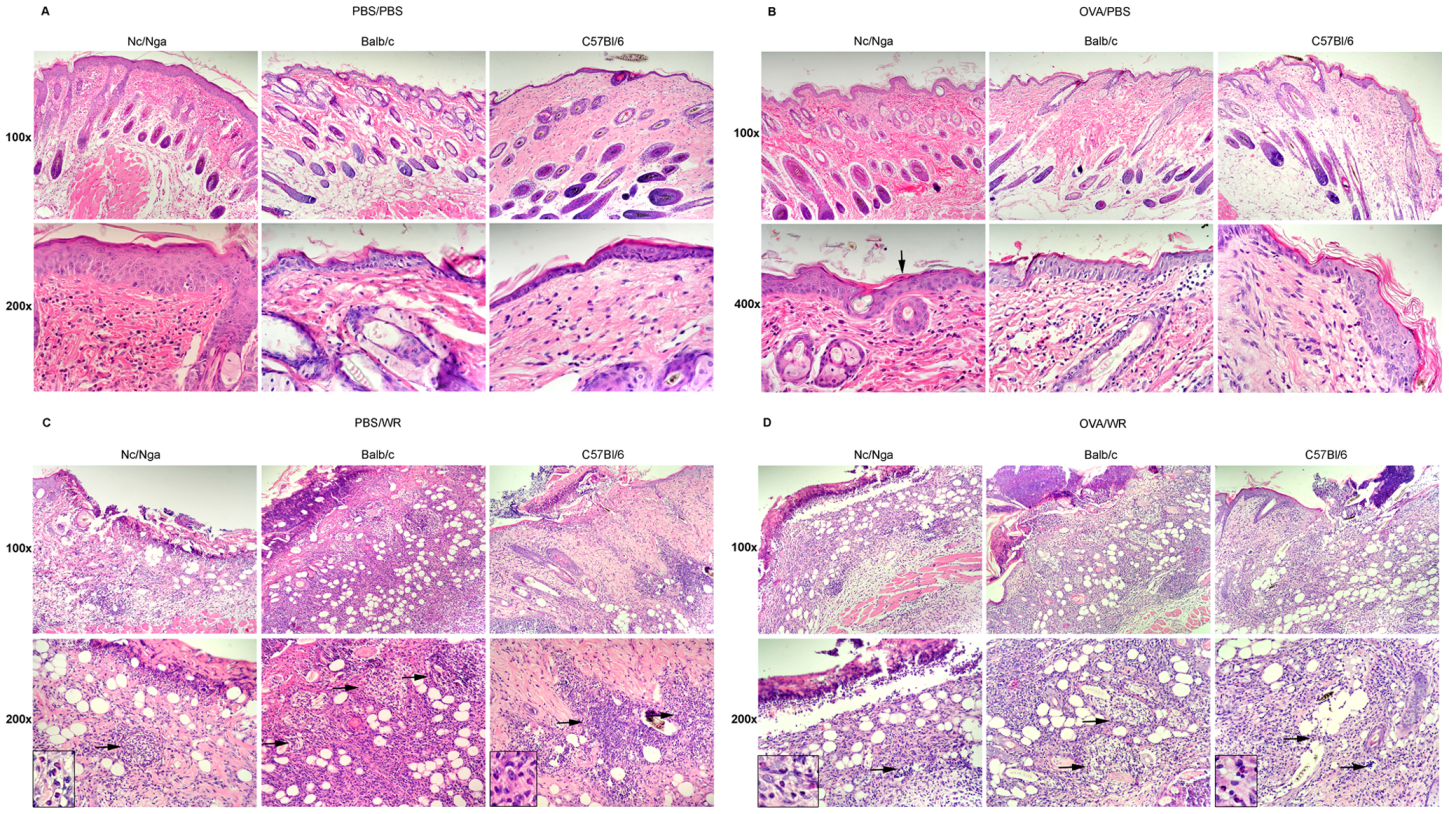
Histological features of the skin of Nc/Nga, Balb/c and C57Bl/6 mice 7 days after mock or WR inoculation by vaccination stamp. Mock- (A, C) and OVA-sensitized (B, D) mice were mock-inoculated with OVA in PBS (A, B) or inoculated with 10^7^ PFU of a purified stock of WR with OVA in PBS (C, D). Hematoxylin-eosin stained skin sections. Original magnification: (A, B) upper panel 100x, lower panel 400x; (C, D) upper panel 100x, lower panel 200x with inserts 400x. (A) Nc/Nga - Mild mixed predominantly mononuclear dermal inflammatory infiltrate. Thickening of epidermis, increased thickness of dermis due to proliferation of the connective tissue. Balb/c - Mild fibrosis and no or minimal mixed inflammation in the dermis. C57Bl/6 - Minimal mixed dermal inflammatory infiltrate in the skin, focal thickening of epidermis. (B) Nc/Nga – Very mild mixed dermal inflammatory infiltrate. Thickening of epidermis and parakeratosis (↓), increased thickness of dermis. Balb/c - Thickening of epidermis, mild fibrosis and mild mixed dermal inflammation. C57Bl/6 - Focal thickening of epidermis, very mild mixed dermal inflammatory infiltrate. (C, D) WR-inoculated mice. In all strains - mixed dermal inflammatory infiltrate with an epidermal erosion/ulcer, covered by a crust. Florid necrotizing folliculitis with high incidence of chromatin debris →, eosinophils found in Nc/Nga, Balb/c and C57Bl/6 mice in about 5, 1 and 3%, respectively – shown in the inserts. Representative results of skin sections of 3-5 animals.

First, we analyzed histological features of the skin of mock- and OVA-sensitized mice after a mock or WR inoculation in hematoxylin-eosin-stained skin sections. Among the mock-inoculated animals (Fig. 2A, B), both mock- and OVA-sensitized Nc/Nga mice displayed most characteristic features of AD, particularly a mild inflammation, thickening of epidermis and dermis all over the skin sections, proliferation of connective tissue and parakeratosis. Balb/c and C57Bl/6 mice also exhibited thickening of epidermis and dermis but only in some regions of the skin sections; a minimal inflammation was more accentuated in OVA-sensitized animals. In comparison with Nc/Nga mice, the AD-like skin changes were generally milder in Balb/c and C57BL/6 mice, being present mostly in OVA-sensitized animals.

The inflammatory changes in the skin of WR-inoculated Nc/Nga, Balb/c and C57Bl/6 mice were qualitatively similar in all strains studied regardless of saline/OVA sensitization (Fig. 2C, D). The skin displayed a necrotizing folliculitis, a high incidence of the apoptotic debris, and a mixed inflammatory infiltrate (polymorphonuclear and mononuclear cells) in dermis. Occasionally, eosinophils, important mediators of allergic responses, were observed in sections of all three mouse strains, constituting about 5, 1 and 3% of inflammatory elements in Nc/Nga, Balb/c and C57Bl/6 mice, respectively (inserts in Fig. 2C, D). The site of inoculation was ulcerated, often covered with a crust. The size of the lesions and skin injury was generally greater in Nc/Nga mice, as characterized by deep ulcerations. In contrast in Balb/c and C57Bl/6 mice, the activity of the inflammation was greater, while the ulcerations were more superficial.

In addition to eosinophils, allergic responses are mediated also by mast cells. Therefore, we have determined number of mast cells in sections parallel to hematoxylin-eosin stained sections before and after WR inoculation using Toluidine blue staining (Table 1). In Nc/Nga mice, the number of mast cells was significantly higher than in Balb/c and C57Bl/6 mice and did not seem to be affected by sensitization with OVA or by WR 7 days p.i. On the other hand, there was a decrease in numbers of mast cells associated with the OVA sensitization in both Nc/Nga and Balb/c mice at the day 0.

**Table 1:** Quantification of mast cells in Toluidine blue-stained skin sections from mock- and OVA-sensitized mice at the day of inoculation and 7 days after mock- or WR-inoculations.

Number of mast cells						
	Day of inoculation		7 days post infection			
	PBS	OVA	PBS/PBS	OVA/PBS	PBS/WR	OVA/WR
Nc/Nga	23.45±1.41	18.65±2.15	16.10±3.50	21.78±7.88	21.63±3.17	22.28±4.45
Balb/c	12.50±1.01***	8.03±0.67***	7.31±1.24^x^	6.07±0.72** a	11.13±1.21**	10.99±2.03* a
C57Bl/6	ND	ND	8.15±3.55	6.53±0.47**	9.23±3.35**	13.56±1.93*

Data represent mean +/− S.E.M.; n = 2–5 mice in each group. ND, not determined. Statistically significant decrease in the number of mast cells compared to analogously sensitized Nc/Nga mice,

x P<0.1,

*P<0.05,

**P<0.01;

***P<0.001. Statistically significant decrease in the number of mast cells in OVA-sensitized Balb/c mice compared to mock-sensitized Nc/Nga mice.

a P<0.05.

Altogether, the histological analyses revealed that skin samples of both mock- and OVA-sensitized Nc/Nga mice displayed the most characteristic features of AD of all mouse strains and treatments. In contrast, only the skin samples of OVA-sensitized Balb/c and C57Bl/6 mice revealed signs of AD that were generally milder than in Nc/Nga mice. The inflammatory changes in the skin after inoculation of WR were qualitatively similar in all strains and treatments, while infiltration with eosinophils and mastocytes, elements typical for allergic responses, was the highest in Nc/Nga mice regardless of sensitization.

#### 1.2. WR spread in the skin

**Figure 3:**
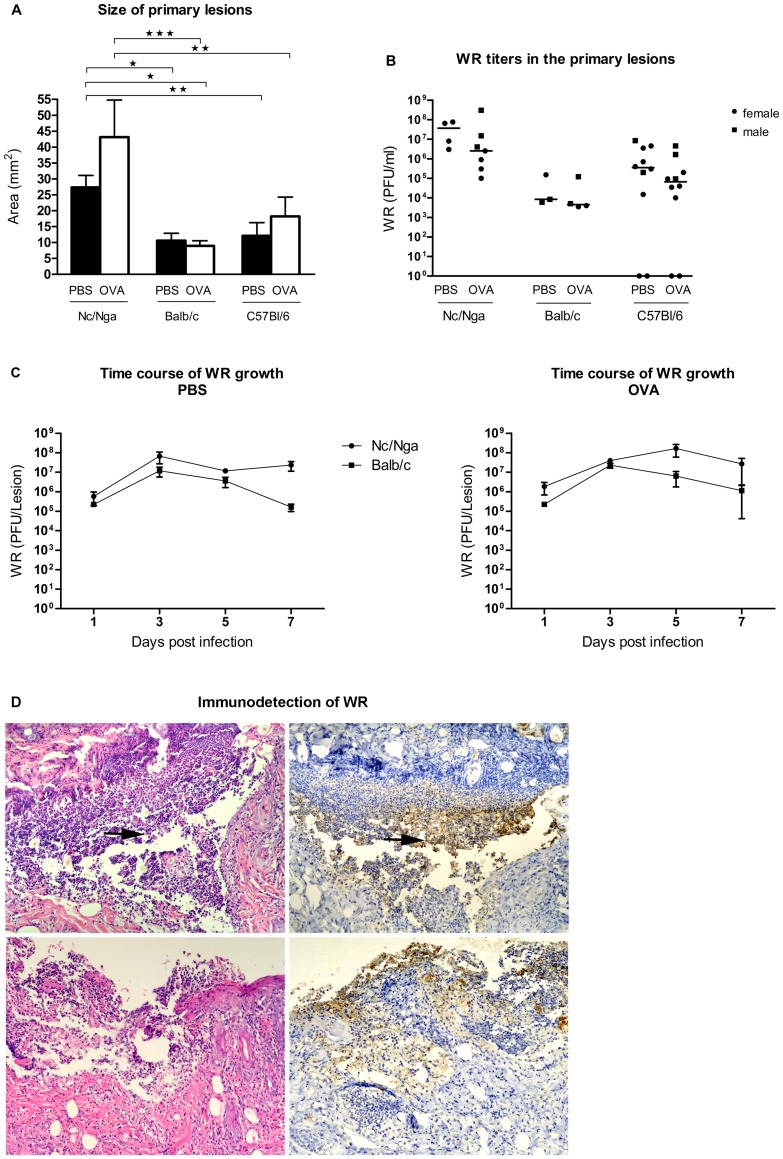
Primary and secondary lesions in the skin of Nc/Nga, Balb/c and C57Bl/6 mice after WR inoculation by vaccination stamp. Mock- and OVA-sensitized mice were inoculated with 10^7^ PFU of a purified stock of WR with OVA in PBS, sacrificed at indicated days p.i, and skin biopsies were taken. (A) Size of primary lesions 7 days p.i. Data represent mean +/− S.E.M. *P<0.05, **P<0.01. Results of 2 experiments with n = 6–9 mice in each group. (B) WR titers (PFU/ml) in the site of WR inoculation 7 days p.i.; median values are indicated. No statistically significant differences. Results of 1–2 independent experiments. (C) Time course of WR growth. Data represent mean +/− S.E.M.; n = 3–12 mice in each group. No statistically significant differences. Results of 3 and 2 independent experiments in Nc/Nga mice and Balb/c mice, respectively. (D) Site of primary inoculation (upper panels) and a satellite lesion (lower panels) in mock-sensitized, WR-inoculated Nc/Nga mice 7 days p.i. Immunohistochemical detection of WR (right), control hematoxylin-eosin staining (left); original magnification 200x. Positive IHC signal (brown) overlaps with the sites of increased apoptosis (→).Representative results of 3 skin sections.

Eczema vaccinatum is characterized by an uncontrolled replication and spread of vaccinia virus throughout the skin apart from the site of inoculation. Therefore, the size of the primary lesions, virus yields and development of satellite lesions can serve as quantitative indicators of WR spread and development of EV. As shown in Fig. 3A, the size of lesions developed in Nc/Nga mice was significantly bigger than in Balb/c and C57Bl/6 mice in both mock- and OVA-sensitized groups. Then, we determined the yields of WR achieved in the inoculation sites of all three mouse strains 7 days p.i. (Fig. 3B), with WR yields in mock-sensitized Nc/Nga mice being about 3 logs higher than in Balb/c and about 1.5 log higher than in C57Bl/6 mice. We have also quantified virus growth in the lesions of Nc/Nga and Balb/c mice in a time course experiment (Fig. 3C). At all time points, virus yields were found higher in Nc/Nga mice, confirming that control of WR replication and clearance were impaired in comparison to Balb/c mice. Nevertheless, the differences among individual strains and treatments presented in Figs. 3B and 3C were not found statistically significant due to a considerable variability.

Further, we quantified the number of satellite lesions, characterizing WR spread in the skin of infected animals. We found the occurrence of satellite lesions very frequently in all Nc/Nga and in OVA-sensitized Balb/c mice (Table 2). In all mice included in this table, the presence of virus in the secondary lesions was proved either by IHC (Fig. 3D) or by virus titration. IHC sections were also used to analyze the presence of apoptotic debris in parallel to hematoxylin-eosin stained skin sections, confirming that apoptosis occurred mostly in cells that stained positive also for WR (Fig. 3D).

**Table 2:** Percentage (%) of Nc/Nga, Balb/c and C57Bl/6 mice with 1 or more satellite lesions 7 days p.i. with WR.

Percentage of mice with satellite lesions				
	PBS/WR		OVA/WR	
	%	n	%	n
Nc/Nga	44	9	56	9
Balb/c	0	10	40	10
C57Bl/6	0	6	0	6

n =  number of mice in each group.

In summary, these results indicate that Nc/Nga mice, both mock- and OVA-sensitized, revealed the largest primary lesions, the highest virus yields and occurrence of eosinophils and mastocytes in primary lesions and the highest occurrence of satellite lesions of all three strains and treatments. Thus, Nc/Nga mice seem to represent a better model of AD/EV than OVA-sensitized Balb/c or C57Bl/6 mice.

#### 1.3. Levels of total IgE and specific antibodies against WR

**Figure 4:**
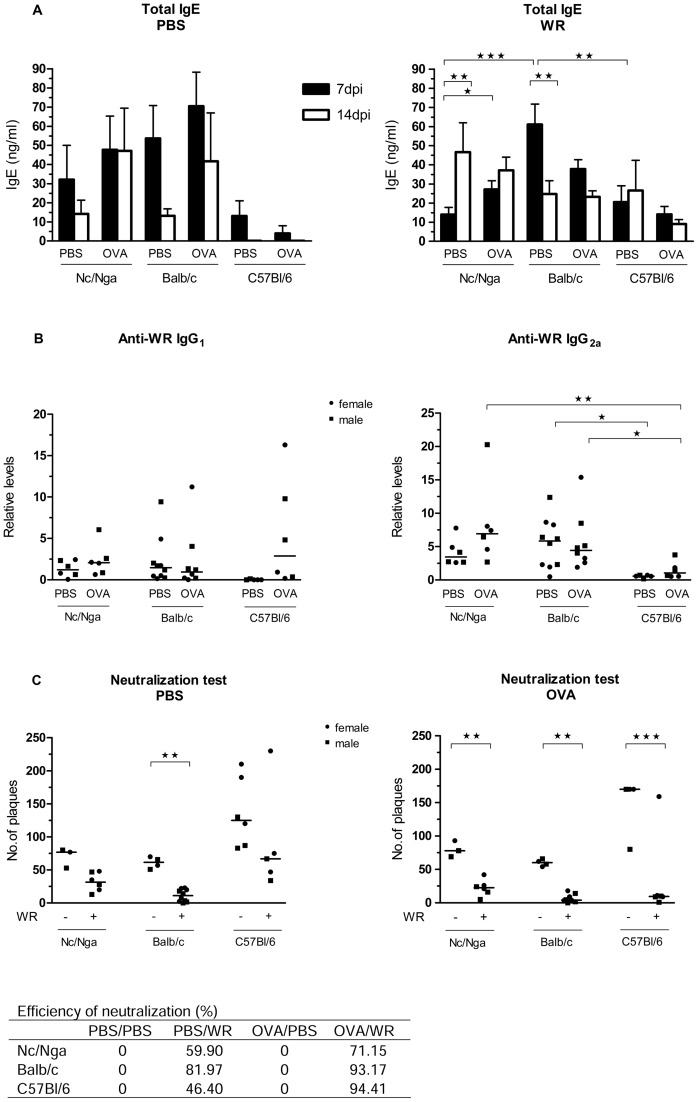
Immune responses after WR inoculation. Mock- and OVA-sensitized Nc/Nga, Balb/c and C57Bl/6 mice were mock-inoculated with OVA in PBS or inoculated with 10^7^ PFU of a purified stock of WR with OVA in PBS, sacrificed 7 and 14 days later, and the levels of antibodies were determined by ELISA. (A) Levels of total serum IgE 7 and 14 days after inoculation expressed as mean +/− S.E.M. Results of 1–4 independent experiments. (B) Relative levels of WR-specific antibodies 14 days after inoculation were determined by ELISA (dilution 1∶1,000); median values are indicated. Results of 2 independent experiments. (C) Neutralization capacity of naive (-WR) and immune (+WR) mice sera 14 days after inoculation expressed as number of plaques detected after a 12-h incubation of the input virus with the individual sera; see Materials and Methods section 8 for experimental details. Median values of the resulting numbers of plaques are indicated in the graphs; the table represents efficiency of neutralization capacity in percentage, where mock-inoculated mice represent 0% of neutralization. Results of 2 independent experiments for each mouse strain. *P<0.05, **P<0.01, ***P<0.001.

In order to further characterize allergic and specific immune responses in the three strains, we determined levels of total IgE antibodies (Fig. 4A) and levels of WR-specific antibodies IgG_1_ and IgG_2a_ as isotypes typical for Th_2_- and Th_1_-skewed responses, respectively (Fig. 4B). Between 7 and 14 days p.i., total IgE decreased in all groups of mock-infected animals, except of OVA-sensitized Nc/Nga mice in which the levels of total IgE remained comparably high at both time points. In WR-inoculated animals, a statistically significant increase in the levels of total IgE was found between 7 and 14 days p.i. in mock-sensitized Nc/Nga mice and a slight, statistically not significant increase in OVA-sensitized Nc/Nga mice again. In contrast in Balb/c mice, total IgE levels decreased in time in both groups, while in C57Bl/6 total IgE levels were low and the changes insignificant. These results suggest that IgE levels increase in time in response to OVA and/or WR in Nc/Nga mice, while they decrease in time in the other strains and treatments.

The production of WR-specific antibodies IgG_1_ and IgG_2a_ was found very low, almost undetectable 7 days after WR-inoculation in all 3 mouse strains studied (data not shown). Fourteen days p.i., the levels of WR-specific antibodies were already detectable, with higher levels found in Nc/Nga and Balb/c strains (Fig. 4B). In C57Bl/6 mice, levels of specific IgG_1_ and IgG_2a_ were very low, with the exception of IgG_1_ levels in OVA-sensitized animals. Yet, the relative levels of IgG_1_ cannot be compared to the levels of IgG_2a_ in the tested samples as the amount of individual IgG_1_ and IgG_2a_ in the standard is unknown. When comparing levels of individual IgG isotypes among the three mouse strains, C57Bl/6 mice can serve as an established control for Th1-dominant responses. However at this time interval, our data do not confirm the higher IgG_1_ levels typical for Th_2_-skewed responses in Nc/Nga and Balb/c mice as it is reported elsewhere.

We have further characterized expression of IL-17A and IFNγ in the skin after WR inoculation in Nc/Nga and Balb/c mice using real-time RT-PCR. IL-17A was previously reported to inhibit immigration of NK and other cell types into the site of infection in AD skin [22] and to promote WR replication [23], while IFNγ reveals immunomodulatory and antiviral properties ([34]). The results summarized in S1 Figure indicate that both cytokines were increased 5 days after WR inoculation in both mouse strains.

#### 1.4. Serum neutralization capacity

The efficiency of humoral immunity can be assessed by determination of total neutralization capacity of the immune sera. Therefore, the sera of mock- and WR-inoculated Nc/Nga, Balb/c and C57Bl/6 mice were used for plaque reduction assays. The control, non-immune sera decreased the input virus about 10-times in all strains, with sera of C57Bl/6 revealing somewhat lower neutralizing capacity. This result reflects the action of complement, as no heat inactivation was performed. Seven days p.i., no sera of WR-inoculated mice studied were able to neutralize WR (data not shown), correlating with the minimal production of WR-specific antibodies 7 days p.i. described above. Fourteen days p.i., sera of all three WR-inoculated mouse strains already revealed a capacity to neutralize WR (Fig. 4C), with the differences being significant in all OVA-sensitized animals and in mock-sensitized Balb/c mice. Importantly, neutralization capacity of WR-inoculated Nc/Nga mouse sera was lower than the one of WR-inoculated Balb/c mouse sera (See table included in Fig.4C).

In summary, neutralization tests indicated a clear difference between immune and non-immune sera, with Nc/Nga mice revealing lower or delayed specific neutralization capacity than Balb/c and OVA-sensitized C57Bl/6 mice.

### 2. Protective effect of MVA and Dryvax immunizations in atopic Nc/Nga mice

Based on the above described results, we have decided to use the atopic Nc/Nga mice, the model of AD susceptible to develop EV after inoculation of replicating WR, to test the efficiency and safety of immunization with a non-replicating vaccinia virus MVA against a lethal challenge with the intra-nasal administration of WR, the surrogate of smallpox. For comparison, we also immunized the mice with the commonly used Dryvax, the original smallpox vaccine associated with various post-vaccination complications.

#### 2.1. Immunization with MVA and Dryvax

The mock-sensitized Nc/Nga mice were mock-immunized with PBS or immunized with Dryvax and MVA t.d. using the vaccination stamp or with MVA i.m. Seven days after the immunization, no lesions were visible at the sites of t.d. administration of MVA, while there were relatively large skin lesions at the sites of t.d. administration of Dryvax; the diameter of the lesions caused by Dryvax was comparable to the lesions produced by WR described above (Fig. 3A). Occasionally, satellite lesions were found in Dryvax-inoculated, mostly male mice, confirming again the risk of VACV spread in the atopic organism. Administration of MVA i.m. did not cause any apparent macroscopic changes. Dryvax was not administered i.m., since it is a lytic virus and the route of administration used in human is t.d. only. Thus, vaccination with MVA seems to be safe, without any signs of spread of the virus.

#### 2.2. Specific antibodies against VACV and serum neutralization capacity

**Figure 5:**
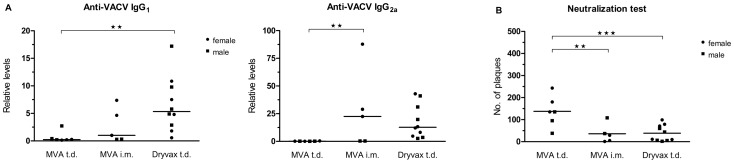
Immune responses of atopic Nc/Nga mice immunized with MVA and Dryvax. Mock-sensitized Nc/Nga mice were inoculated using a vaccination stamp trans-dermally (t.d.) or intra-muscularly (i.m.) with 10^8^ PFU of a purified stock of MVA or t.d. with 10^7^ PFU of a purified stock of Dryvax, and sacrificed 3 weeks later. (A) Levels of total serum IgG_1_ and IgG_2a_ antibodies specific to VACV were determined by ELISA (dilution 1∶1,000); median values are indicated. Results of 2 independent experiments. (B) Neutralization capacity of mice sera expressed as number of plaques detected after a 12-h incubation of the input virus with the individual sera; see Materials and Methods section 6 for experimental details. Median values of the resulting numbers of plaques are indicated. Results of 1–2 independent experiments. *P<0.05, **P<0.01.

In order to characterize the immune responses to the individual immunization protocols, the levels of VACV-specific IgG_1_ and IgG_2a_ and the neutralization capacity were determined three weeks after the immunization, just before exposure to the lethal dose of WR. As shown in Fig. 5A, immunization with Dryvax resulted in fair amounts of antibodies of both isotypes, while MVA administered t.d. yielded non-detectable or very low amounts of both isotypes. MVA administered i.m. resulted in low levels of VACV-specific IgG_1_ and in somewhat higher levels of IgG_2a_. Additionally performed neutralization tests (Fig. 5B) inversely correlated with the levels of IgG_2a_ antibodies. In summary, after MVA-immunization t.d., the animals revealed lower humoral responses compared to MVA i.m. and Dryvax-immunized ones.

#### 2.3. Lethal intranasal challenge with VACV strain WR

**Figure 6:**
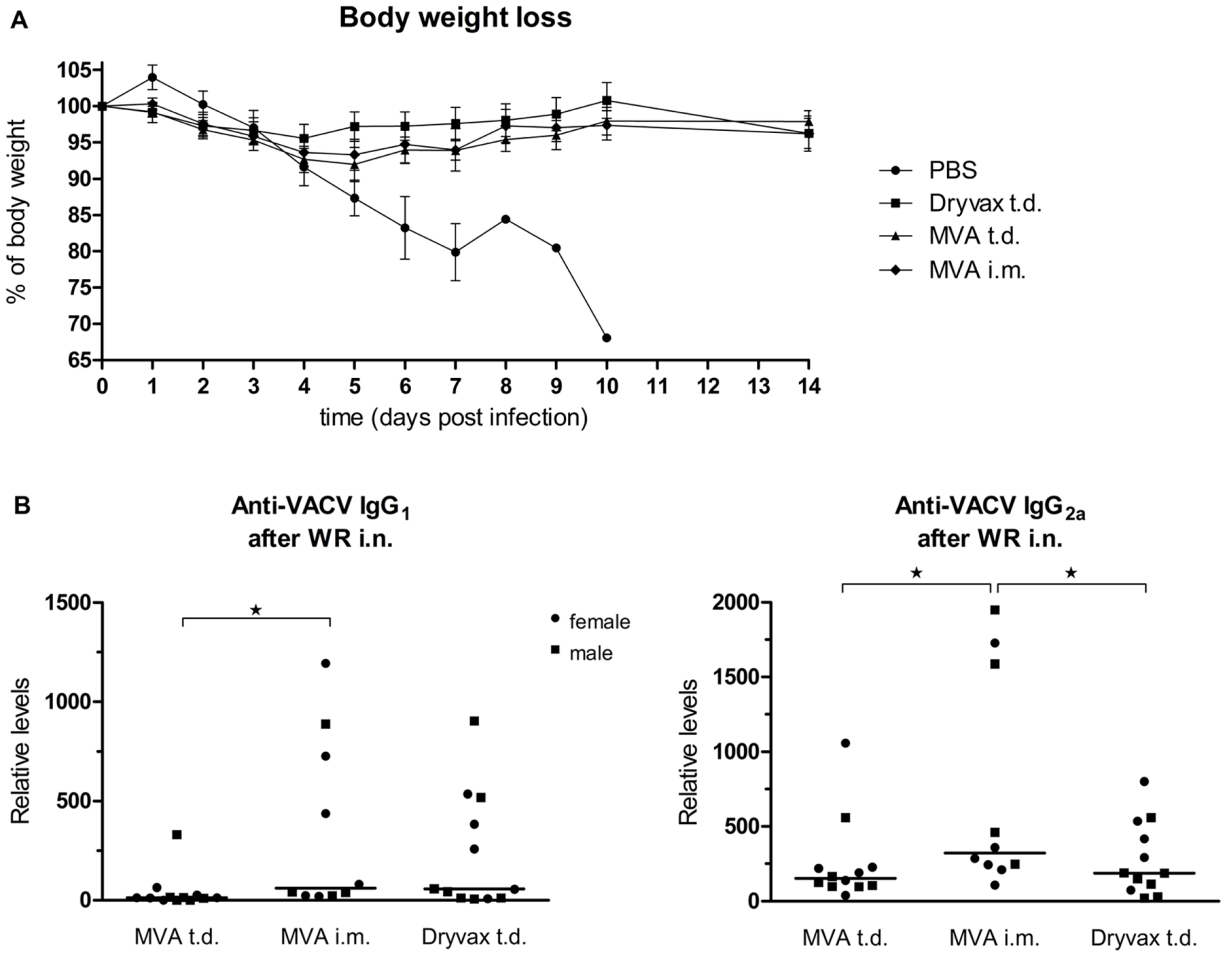
Survival of atopic Nc/Nga mice immunized with MVA or Dryvax after a lethal intranasal challenge with VACV strain WR. (A) Body weight changes of mock-immunized (PBS), MVA- or Dryvax-immunized Nc/Nga mice after lethal i.n. challenge with WR. Results are expressed as mean +/− S.E.M. At day 10, all mock-immunized mice were spontaneously dead. Results of 2 independent experiments performed for each virus strain together with the PBS controls. (B) Levels of total serum IgG_1_ and IgG_2a_ antibodies specific to VACV determined 14 days after a lethal challenge with VACV strain WR determined by ELISA in sera dilutions 1∶10,000–1∶20,000 and 1∶100,000–1∶200,000 for IgG_1_ and IgG_2a_, respectively, and expressed as relative values; median values are indicated. Results of 2 independent experiments.

In order to test the protective effect of immunization with MVA and Dryvax in atopic animals in vivo, the animals were challenged with a lethal i.n. dose of WR, the surrogate of smallpox. In spite of the variation in immune responses raised by the two virus strains and different ways of immunization, results of two independent experiments clearly demonstrated that a vast majority of MVA-immunized mice as well as all Dryvax-immunized mice were able to survive a lethal dose of VACV strain WR administered i.n. (Table 3). The surviving animals did not display any changes in behavior or moving, any significant weight losses (Fig. 6A) or signs of a serious illness. In contrast, all the mock-immunized controls (with PBS administered t.d.) succumbed to the infection. They displayed initially a bristled hair, later an enlargement of abdomen and difficult breathing; nevertheless they died spontaneously before meeting criteria for the humane endpoint defined in the study protocol. The post-mortem dissection revealed inflation of the gut, saddle macroscopic changes in color of lungs (somewhat hemorrhagic) and very small, exhausted spleens. WR titers were determined at the day of death in mock-immunized mice. The virus was commonly found in lungs, with titers ranging from 10^1^ to 10^7^ PFU/organ. In some mice, the virus was found also in liver, spleen and kidneys, with the highest virus yields usually in lungs at day 5 p.i., occasionally in liver. In MVA-immunized mice that succumbed to the infection, no virus was found in any organ. The exact cause of death was not further explored. In the surviving, previously immunized animals, virus titers were not determined when they were sacrificed humanely 14 days after the lethal infection as the virus is never detected at this time interval. When sacrificed, they displayed pale, pink lungs and the increased size of spleen.

**Table 3:** Survival of atopic Nc/Nga mice immunized with MVA or Dryvax after a lethal intranasal challenge with VACV strain WR.

Survival of immunized mice				
	PBS	MVA	MVA	Dryvax
	t.d.	t.d.	i.m.	t.d.
Survival	0/17	12/13*	10/12*	12/12
Survival (%)	0	92	83	100
Mean day of death ± S.D.	6±0.5	6	10	-
Splean ± S.D. (mg)	52±5†	132±10	118±14	109±5
Lungs ± S.D. (mg)	145±12‡	168±6	171±5	186±9

Mean day of death and weights of spleens and lungs at death or at sacrification 14 days after the challenge are indicated. SD, standard deviation;

*, dead mice were males;

† , weight of normal spleens of age-matched mice is 70–80 mg;

‡ , weight of normal lungs of age-matched mice is 140–170 mg. Results of 2 independent experiments performed for each virus strain together with the PBS controls.

Additionally, we have determined levels of VACV-specific IgG_1_ and IgG_2a_ antibodies in the surviving animals. As summarized in Fig. 6B no significant differences in the levels of either IgG_1_ or IgG_2a_ antibodies were found, with the levels of both isotypes being about 10–100-times higher than those determined just before the i.n. challlenge. This increase might suggest that the challenge virus replicated in the organism but its replication was controlled by specific immune responses of Nc/Nga mice.

In summary, immunization of atopic Nc/Nga mice with a single dose of MVA administered either t.d. or. i.m., resulted in protection of most animals against a lethal poxvirus challenge without any serious illness.

## Discussion

In this work, we compared characteristics of atopic dermatitis and immune responses of three different mouse strains, Nc/Nga, Balb/c and C57Bl/6, towards inoculation of VACV strain WR into the skin, concluding that Nc/Nga mice are the most suitable model of eczema vaccinatum. Consequently, we used these mice to prove the ability of the non-replicating MVA to induce a protective immunity against a lethal i.n. challenge with VACV strain WR, the surrogate of smallpox. To our knowledge, this is the first report proving efficiency of MVA immunization against a lethal poxvirus infection in vivo in an atopic organism.

Based on our results, the skin changes of Nc/Nga mice meet most characteristics of an atopic skin. The histological analysis showed that the pathological changes were similar in both mock- and OVA-sensitized skins of Nc/Nga mice, while they were less pronounced in Balb/c and C57Bl/6 mice, mostly after the sensitization with OVA. The authors of this EC sensitization protocol, Spergel and Jin et al. [19], [20], reported development of skin allergic inflammation after TS and an epicutaneous sensitization with OVA in Balb/c mice, and also Scott et al. [24] reported development of allergic inflammation with eosinofilia and CD4+ cells infiltration in skin of Balb/c mice after EC application of OVA together with TS. In Nc/Nga mice, eczematous lesions were reported to be effectively induced also by other allergens. The group of Kawakami et al. [22], [35] induced allergic inflammation in Nc/Nga mice using dust mites and Staphylococcal enterotoxin B. Accordingly, we have tested dust mites and household fungi with results similar to OVA treatment (data not shown).

After inoculation of WR, both mock- and OVA-sensitized Nc/Nga mice developed large skin lesions together with satellite lesions that could be easily distinguished from the site of inoculum. The primary skin lesions in other strains and groups were much smaller and satellite lesions could be found only in OVA-sensitized Balb/c mice. In Nc/Nga mice, use of lower doses of WR and Dryvax was also tested, resulting in even larger primary lesions and higher occurrence of secondary lesions, regardless of the addition of OVA into the virus inoculum (unpublished results). Indeed, this outcome is in agreement with previous observations in human vaccinees [36]. Development of satellite lesions were observed also by others, but the presence of VACV was not proven [23], [24] or their occurence was low [22].

In human, AD is often associated with elevated IgE production, with total serum IgE being increased in about 80% of AD (extrinsic AD; [13]). In Nc/Nga mice kept in conventional housing, serum IgE levels were found increased compared to those kept in SPF conditions [18], [37], [38], but IgE production was described to be dispensable for atopic changes in the skin of Nc/Nga mice [20], [39]. Accordingly, we observed the atopic changes of the skin histologically, but we found relatively low levels of IgE in mock-sensitized, mock-inoculated Nc/Nga mice and also relatively low levels of WR-specific IgG_1_, described to be elevated in epicutaneously sensitized and WR-infected Balb/c mice [24]. Our results of IFNγ expression (S1 Figure) are compatible with those of Yagi et al. [39] who demonstrated that AD pathology in Nc/Nga was IgE/Th2-independent and that the eczematous symptoms were caused by the IFNγ-favored environment. Also, Mammesier et al. [40] proposed that the allergic inflammatory response would not be due to a Th1/Th2 imbalance, but rather to a Treg deficiency.

Interestingly, in Nc/Nga mice, total serum IgE levels were increasing between 7 and 14 days post infection with WR (with the statistical significance only in mock-sensitized Nc/Nga mice). We suggest that this IgE increase in Nc/Nga mice might be due to constantly high amounts of mast cells in the skin of these mice that might lead to the release of mediators like heparin and histamin. Heparin can activate interferon-inducible dsRNA-activated protein kinase R, PKR [41], which is involved in IgE class switching and plays a role in IgE production [42]. It was reported, that VACV inhibits PKR activation through the E3L protein [43], [44], but in atopic Nc/Nga mice, this inhibition might be overcome by heparin; thus PKR can be still activated and contribute to IgE increase.

Histological and immunohistochemical analyses further revealed the presence of chromatin debris at the sites of VACV inoculation, indicating a DNA fragmentation during the ongoing apoptosis. These results thus suggest that VACV inoculation into the skin might result in apoptosis rather than necrosis of infected cells. It has been previously established that VACV, typically considered as a lytic virus, causes apoptosis of various immune cell types (DC, macrophages, B-cells; [33], [45], [46]. Further, we have recently demonstrated, that VACV strain WR caused either apoptosis or necrosis in different epithelial cell lines and that vaccination strains Dryvax and Praha caused apoptosis in two epithelial cell lines tested [29]. Further, different recombinants of VACV strain WR caused apoptosis in human fibroblast cell line NIH 3T3 (unpublished results). The type of cell death and presence of various PAMP's and DAMP's [47] are critical for the type of immune responses raised, and thus for better understanding to the post-vaccination complications. It is therefore important to closely characterize the type of cell death induced by VACV *in vivo* in the individual cell types.

The specific immunity of Nc/Nga, Balb/c and C57Bl/6 mice towards VACV strain WR was characterized by production of WR-specific IgG_1_ and IgG_2a_ and by the capacity of mouse sera to neutralize the virus infectivity. The antibody responses were detectable only 14 days p.i. and they were relatively weak in all strains, as the peak levels are expected only later. Additionally, C57Bl/6 mice were reported to express the IgG_2c_ isotype instead of IgG_2a_ [48]. On the other hand, neutralization tests indicated a clear difference between immune and non-immune animals, with Nc/Nga mice revealing lower or delayed specific neutralization capacity than Balb/c mice.

The above discussed results showed that Nc/Nga mice inoculated with VACV strain WR fulfill characteristics of a model of eczema vaccinatum independently of OVA sensitization, suggesting that they are the model of choice for such studies. In contrast, in Balb/c and C57Bl/6J mice, sensitization with OVA was necessary. Nevertheless, Balb/c mice sensitized with OVA and other allergens have been successfully used in studies of EV [23], [24].

MVA, attenuated VACV that does not replicate in mammals, seems to be a safe option for inducing antipoxviral immunity, especially in atopics that cannot be vaccinated with replicating VACV. Previously, MVA effect was demonstrated in atopic, OVA-sensitized Balb/c mice, in which MVA immunization prevented the appearance of lesions after a subsequent inoculation of WR into the skin [49]. MVA also seems to induce good immune responses in blood tests in human atopics [50], but the efficacy cannot be tested in vivo. Therefore, we used the atopic Nc/Nga mice for testing efficiency and safety of MVA immunization against a lethal poxviral challenge and compared it with Dryvax, the old smallpox vaccine associated with post-vaccination complications. We have used immunization with a single dose of t.d. or i.m. administered MVA and a single dose of t.d. administered Dryvax. The immunization with Dryvax protected 100% of mice, but it should be emphasized that these mice revealed relatively large skin lesions and formation of satellite lesions after the virus inoculation, further confirming an increased risk of development of eczema vaccinatum in AD individuals. On the other hand, immunization with non-replicating MVA that is safe even for atopics and that did not lead to development of any lesions detectable one week after immunization, led to a substantial, but incomplete survival of the immunized animals. The number of MVA-immunized animals that succumbed to the lethal challenge with WR was too low to indicate any significant differences between the t.d. and i.m. immunizations. However it appears that most animals that probably did not develop any detectable levels of antibodies after a single dose of MVA were still able to successfully defeat the lethal challenge with VACV strain WR. These results suggest that either the production of specific antibodies amplified quickly to sufficient levels after the second encounter with VACV or that the control of virus spread was mediated mostly by cellular immunity. Nevertheless both wild-type, replicating strains of VACV and MVA were previously shown to induce apoptosis of dendritic cells and thus affect their antigen-presenting function as well as other immune responses [46], [51], [52]. In any case, regardless of the exact mechanisms involved, our results prove the usefulness and efficiency of the immunization with MVA in an atopic organism. Probably, this efficiency could be further improved if a protocol consisting of several consecutive doses of MVA was used.

For obvious reasons, the mounting of protective responses against smallpox cannot be tested in human, but the re-introduction of vaccination of the general population still has been considered [53]. Our work thus supports usefulness and efficacy of MVA-based vaccines against lethal poxviral infection also in an atopic organism.

## Supporting Information

S1 Figure **Expression of IL-17A and IFNγ cytokines in the skin after WR inoculation.** Skin biopsies were taken from mock- and OVA-sensitized Nc/Nga and Balb/c mice 5 days after mock-inoculation with OVA in PBS (PBS) or after inoculation with 10^7^ PFU of a purified stock of WR with OVA in PBS (WR). Expression of individual cytokines in the skin was determined by real-time RT-PCR and standardized to GAPDH (expressed as ratio cytokine/GAPDH ×10^5^). Data represent mean +/− S.E.M.; number of animals in Nc/Nga and Balb/c groups: 3 and 4, respectively. *P<0.05, **P<0.01, *** P<0.001. **S1 Figure Methods**: *Determination of cytokine expression by real-time RT-PCR*. Biopsies of skin lesions were stored in RNA*later* RNA Stabilization Reagent (Qiagene) or Allprotect Tissue Reagent (Qiagene). RNA was isolated after homogenization of the skin samples in RNA Blue (Top-Bio, Czech Republic) using manufacturer's protocol. Purified RNA was resuspended in RNase-free water with the addition of RNase inhibitor RiboLock (Fermentas). RNA concentration and purity were determined by measuring the absorbance at 260 and 280 nm, respectively, using UV spectrophotometer BioPhotometer (Eppendorf AG). RNA was treated with RNase-free DNase (Fermentas) in the presence of RiboLock and quantification of RNA of interest was performed by *Power* SYBR Green RNA-to-C_T_ *1-Step* Kit (Applied Biosystems) using Applied Biosystems 7300 Real-time PCR System according to manufacturer's protocol. Quantification of each RNA was performed in duplicate together with GAPDH using 50 ng of RNA in 20 µl reactions and 40 cycles. Specific primers for individual genes were described previously or newly designed using Primer-BLAST (http://www.ncbi.nlm.nih.gov/tools/primer-blast/): Mu GAPDH cDNA forward 5′–CGGTGCTGAGTATGTCGTGGA–3′, reverse 5′–GGCAGAAGGGGCGGAGATGA–3′ [54]; Mu IL-17A cDNA forward 5′–GGACTCTCCACCGCAATGAA–3′, reverse 5′–TTTCCCTCCGCATTGACACA–3′; Mu IFNγ cDNA forward 5′–TGGCATAGATGTGGAAGAAAAGAG–3′, reverse 5′–TGCAGGATTTTCATGTCACCA–3′ [55].
(TIF)
