# Supplementary figures and images for: Development of Eczema Vaccinatum in Atopic Mouse Models and Efficacy of MVA Vaccination against Lethal Poxviral Infection
Source: PLoS One. 2014 Dec 8;9(12):e114374. doi: 10.1371/journal.pone.0114374 (PMC4259321; doi:10.1371/journal.pone.0114374)

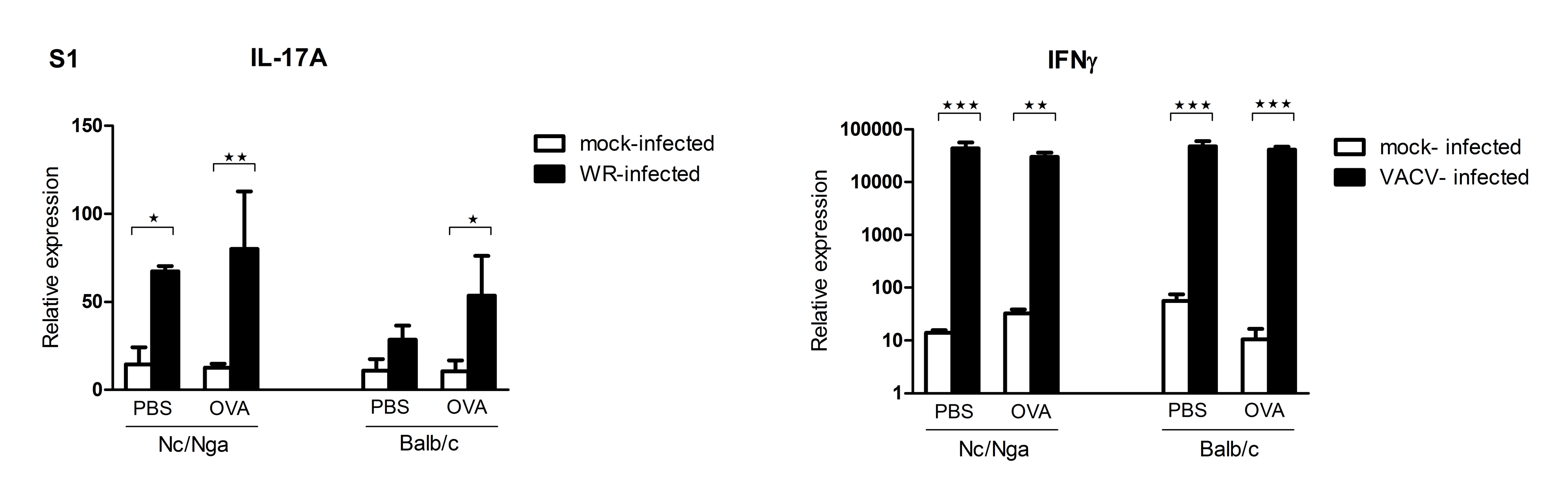

Supplement: S1 Figure — Expression of IL-17A and IFNγ cytokines in the skin after WR inoculation. Skin biopsies were taken from mock- and OVA-sensitized Nc/Nga and Balb/c mice 5 days after mock-inoculation with OVA in PBS (PBS) or after inoculation with 107 PFU of a purified stock of WR with OVA in PBS (WR). Expression of individual cytokines in the skin was determined by real-time RT-PCR and standardized to GAPDH (expressed as ratio cytokine/GAPDH ×105). Data represent mean +/− S.E.M.; number of animals in Nc/Nga and Balb/c groups: 3 and 4, respectively. *P<0.05, **P<0.01, *** P<0.001. S1 Figure Methods: Determination of cytokine expression by real-time RT-PCR. Biopsies of skin lesions were stored in RNAlater RNA Stabilization Reagent (Qiagene) or Allprotect Tissue Reagent (Qiagene). RNA was isolated after homogenization of the skin samples in RNA Blue (Top-Bio, Czech Republic) using manufacturer's protocol. Purified RNA was resuspended in RNase-free water with the addition of RNase inhibitor RiboLock (Fermentas). RNA concentration and purity were determined by measuring the absorbance at 260 and 280 nm, respectively, using UV spectrophotometer BioPhotometer (Eppendorf AG). RNA was treated with RNase-free DNase (Fermentas) in the presence of RiboLock and quantification of RNA of interest was performed by Power SYBR Green RNA-to-CT 1-Step Kit (Applied Biosystems) using Applied Biosystems 7300 Real-time PCR System according to manufacturer's protocol. Quantification of each RNA was performed in duplicate together with GAPDH using 50 ng of RNA in 20 µl reactions and 40 cycles. Specific primers for individual genes were described previously or newly designed using Primer-BLAST (http://www.ncbi.nlm.nih.gov/tools/primer-blast/): Mu GAPDH cDNA forward 5′–CGGTGCTGAGTATGTCGTGGA–3′, reverse 5′–GGCAGAAGGGGCGGAGATGA–3′ [54]; Mu IL-17A cDNA forward 5′–GGACTCTCCACCGCAATGAA–3′, reverse 5′–TTTCCCTCCGCATTGACACA–3′; Mu IFNγ cDNA forward 5′–TGGCATAGATGTGGAAGAAAAGAG–3′, reverse 5′–TGCAGGATTTTCATGTCACCA–3′ [5 [file pone.0114374.s001.tif]
